# Simplified models of the symmetric single-pass parallel-plate counterflow heat exchanger: a tutorial

**DOI:** 10.1098/rsos.171617

**Published:** 2018-03-21

**Authors:** William F. Pickard, Barbara Abraham-Shrauner

**Affiliations:** Department of Electrical and Systems Engineering, Washington University, St Louis, MI 63130, USA

**Keywords:** counterflow, conjugate-Graetz problem, effectiveness, heat exchanger, number of heat transfer units, parallel plate

## Abstract

The heat exchanger is important in practical thermal processes, especially those of (i) the molten-salt storage schemes, (ii) compressed air energy storage schemes and (iii) other load-shifting thermal storage presumed to undergird a Smart Grid. Such devices, although central to the utilization of energy from sustainable (but intermittent) renewable sources, will be unfamiliar to many scientists, who nevertheless need a working knowledge of them. This tutorial paper provides a largely self-contained *conceptual* introduction for such persons. It begins by *modelling* a novel quantized exchanger,^1^ impractical as a device, but useful for comprehending the underlying thermophysics. It then reviews the one-dimensional steady-state idealization which demonstrates that effectiveness of heat transfer increases monotonically with (device length)/(device throughput). Next, it presents a two-dimensional steady-state idealization for plug flow and from it derives a novel formula for effectiveness of transfer; this formula is then shown to agree well with a finite-difference time-domain solution of the two-dimensional idealization under Hagen–Poiseuille flow. These results are consistent with a conclusion that effectiveness of heat exchange can approach unity, but may involve unwelcome trade-offs among device cost, size and throughput.

## 1. Introduction

A heat exchanger is a passive device through which two streams of liquid, separated by a partition, are passed for the purpose of transferring heat energy from one stream to the other. They are common in HVAC (heating, ventilating and air conditioning), petroleum refining and especially in the cooling of internal combustion engines where hot antifreeze from the engine block exchanges its heat with cooler ambient air at the radiator. Moreover, solar thermal electrical generating facilities are frequently constructed to include provision for the storage of sensible heat, so that generation can be extended into the evening hours; recovery of this stored thermal energy to make steam requires, of course, heat exchangers. For the more common applications, exchanger technology can be described as well developed [1–3], and exchanger design is commonly relegated to proprietary software [4]; moreover, the plate heat exchanger has been reviewed in some detail [5] (table 1).

**Table 1.** Nomenclature.

symbol	units	comments
*Latin*		
*a*	m	Half-thickness of the membrane separating the two liquid streams.
*A*	m^2^	Area of the cross-section of a liquid stream carrying heat. A=2d[2h−a].
A	—	A locally defined constant.
*B*	—	An integer specifying the number of domains in the two spatial dimensions. Used in the FDTD (finite-difference time-domain) calculation.
C	—	A locally defined constant.
*d*	m	Half-depth in the *x*-direction of a *z*-ward travelling rectangular stream of liquid.
D	m^2^ s^–1^	Thermal diffusivity of the moving fluid in a heat exchanger. This parameter can be expanded as D=θ/[γρ]. Its numerical value most commonly lies in the range (10^−8^, 10^−4^); for water it is approx. 140 × 10^−9^.
*f*(*υ*)	—	A generic velocity profile across a moving liquid stream.
h	m	Half-height in the *y*-direction of a rectangular pipe with walls of zero thickness, which surrounds a *z*-ward travelling rectangular stream of liquid.
*L*	m	The half-length of finite linear counterflow heat exchanger.
*m*	—	An index for the *υ*-direction during an FDTD calculation. 1 ≤ *m* ≤ 2*B* + 1. *υ* = [*m *– (1 + *B*)]/*B*.
*M*	mol s^–1^	Molar flux rate of a particular stream of liquid through the exchanger.
M	kg mol^–1^	Molar-specific weight of the exchanger liquid.
*n*	—	An index for the *ζ*-direction during an FDTD calculation. 1 ≤ *n* ≤ 2*B* + 1. *ζ* = [*n *– (1 + *B*)]/*B*.
$\tilde{n}$	—	An index used to denote the bolus pairs in an ideal quantized exchanger.
$\tilde{N}$	—	The number of bolus pairs in an ideal quantized exchanger.
*N*	—	Number of warm–cool pairs in a parallel plate exchanger.
NTU	—	Number of heat transfer units, a figure of merit of the exchanger.
*p*	—	A non-negative integer 0, 1, 2, 3, …
***R***	—	A Reynolds number here defined as ***R*** = *Uρh*/*η*
ş	K	A ‘laboratory’ temperature which can be used to characterize the quantity of sensible heat associated with a minute volume of experimental liquid.
$\hat{s}$	°C or K	The *symmetrized* temperature of a liquid stream, defined as $\hat{s}=ş+\Delta$.
*s*	—	The *normalized* symmetrized temperature of a liquid stream, defined as $s=\hat{s}/{\hat{S}}_{0}$.
${ş}_{w,i}$	K	The temperature of a perfectly mixed ‘warm’ liquid in its pipe just outside the influx port on a heat exchanger.
${ş}_{c,i}$	K	The temperature of a perfectly mixed ‘cool’ liquid in its pipe just outside the influx port on a heat exchanger.
${ş}_{w,e}$	K	The temperature of a perfectly mixed ‘warm’ liquid in its pipe just outside the efflux port on a heat exchanger.
${ş}_{c,e}$	K	The temperature of a perfectly mixed ‘cool’ liquid in its pipe just outside the efflux port on a heat exchanger.
$\hat{S}$	°C or K	$\hat{S}=ş+\Delta .$
${\hat{S}}_{0}$	°C or K	${\hat{S}}_{0}=\frac{1}{2}[{ş}_{w,i}-{ş}_{c,i}]={ş}_{w,i}+\Delta =-\{{ş}_{c,i}+\Delta \}.$
*S*	—	$\hat{S}/{\hat{S}}_{0}$
*S*_w,e_	—	A spatially averaged dimensionless efflux temperature defined by equation (4.3*b*).
S	—	A locally defined constant.
*t*	s	The time variable in a non-steady-state heat transfer problem.
*U*	m s^–1^	The mean (or nominal) liquid velocity within a particular stream of the exchanger.
V	m^3 ^mol^–1^	Molar volume of an exchanger liquid.
*z*	m	The axial direction of flow in a rectangular coordinate system describing an exchanger (figure 3).
*Greek*		
*γ*	J kg^–1 ^K^–1^	Specific heat of the exchanger liquid; for water its value is approximately 4190.
*δτ*	—	Size of the dimensionless time step for each iteration of an FDTD calculation.
Δ	°C or K	An offset of heat exchanger temperature, defined as $\Delta =-\frac{1}{2}[{ş}_{w,i}+{ş}_{c,i}]$.
*ε*	—	The thermal *effectiveness* of heat transfer from (or to) a liquid stream in a heat exchanger.
*ζ*	—	A dimensionless length defined by *z* = *Lζ*.
*η*	Pa s	The dynamic viscosity.
*θ*	W m^–1 ^K^–1^	Thermal conductivity of an exchanger liquid; for water its value is approximately 0.61.
*κ*	W m^–1^ K^–1^	Thermal conductivity of the membrane separating two exchanger streams.
*Λ*	—	A dimensionless constant defined by $\Lambda^{2}=Uh^{2}/(LD)$.
*ρ*	kg m^–3^	The density of the liquid in the exchanger; for water its value is approximately 1000.
*τ*	s	A dimensionless time defined by $\tau =Dt/h^{2}$.
*y*	—	A dimensionless length defined by *y* = h*υ*.
*ω_p_*_–1,*p*_	—	The increase in effectiveness as NTU is stepped from (*p *– 1) to *p*.
*Ω*	m^–1^	A device parameter for a one-dimensional counterflow heat exchanger Ω=κd/[MMγa].
*subscript and superscript*		
c	—	A subscript used to denote the ‘cool’ liquid stream in a heat exchanger.
e	—	A subscript used to denote *efflux* from a heat exchanger.
i	—	A subscript used to denote *influx* to a heat exchanger.
w	—	A subscript used to denote the ‘warm’ liquid stream in a heat exchanger.

Exchangers may, however, be due for a renaissance because, with the growing emphasis on energy efficiency and sustainability, a radically different energy infrastructure may have to be developed. For example, despite intense research since the dawn of the Atomic Era, neither controlled fusion [6] nor generally acceptable permanent storage of long-lived fission waste have been developed [7]. In turn, this has heightened perceptions that humankind may have to employ wind and/or direct insolation as a principal source of its supply of sustainable energy: *both are intermittent*. Perforce, metropolitan areas that desire a temporally trustworthy supply of energy must engage in storage of energy—storage which, metropolis by metropolis, is presumably so massive as to be best measured in terms of gigawatt-days.^2^ In turn, storage of sustainable energy on that scale is reputed to mean storage either (i) by pumped hydro schemes or (ii) by advanced adiabatic compressed air energy storage (AA-CAES) [8]. The latter, however, depends in an essential fashion upon counterflow heat exchangers [9] if it is to operate with acceptable energy efficiency. And the exchangers needed for gigawatt-day storage schemes must be of a volumetric throughput and effectiveness of thermal exchange which may prove challenging to present technology. We feel that the unfamiliarity of such devices to virtually all policy-makers and to most scientists, make desirable a ‘simple’ introduction to counterflow heat exchangers. To this end, we introduce in §2, a quantized steady-state exchanger which real freshmen have indeed found to be intuitively simple. In §3, we reprise a one-dimensional exchanger which, in the steady state, permits both analytic solution and ready comprehension. In §4, we tackle two-dimensional exchangers. The quantitation of such devices, even when they are heavily idealized, turns out to be of discouraging complexity, including but not limited to unfamiliar eigenfunction expansions on a rectangle. Therefore, the mathematical arcana have been banished to three appendices. Nevertheless, the selected results we present do indicate clearly that scaling up exchanger-throughput to the high-effectiveness gigawatt-day levels prospectively needed by grid-sized long-term compressed air energy storage could be daunting in the extreme [10].

**Figure 1.**
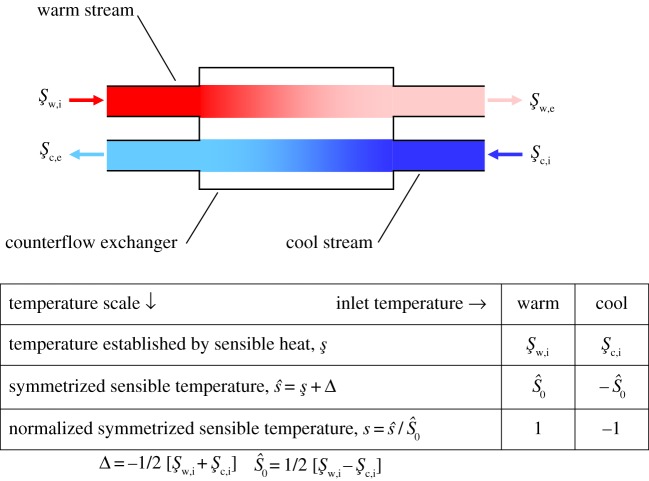
Schematic of a real heat exchanger in steady-state operation. For both warm (red) and cool (blue) streams, the temperatures quoted are just outside the respective ports; the saturation of stream colour diminishes passing through the exchanger to symbolize the heat transfer. The ‘laboratory’ temperature scale, denoted by ş, measures temperature in Celsius-sized degrees with respect to an unspecified zero. The laboratory scale can be transformed to a *symmetrized* scale $\hat{s}=ş+\Delta =ş-1/2[{ş}_{w,i}+{ş}_{c,i}]$ in which the warm inlet temperature is ${\hat{S}}_{0}=1/2[{ş}_{w,i}-{ş}_{c,i}]$ and the cool inlet temperature is –${\hat{S}}_{0}$. And, finally, the symmetrized scale can be divided by ${\hat{S}}_{0}$ to yield a normalized scale $s=\hat{s}/{\hat{S}}_{0}$.

If a heat exchanger is adiabatic, heat is conserved between the two liquid streams and none is exchanged with the exchanger's ambient. If the flow directions of the two streams are predominantly antiparallel, the exchanger is described as ‘counterflow’ (or sometimes as ‘countercurrent’). Consider the generic single-pass counterflow exchanger shown schematically in figure 1. A ‘warm’ influx stream at temperature ${ş}_{w,i}$ enters at the left and runs past a predominantly antiparallel ‘cool’ influx stream at temperature ${ş}_{c,i}$ which enters at the right: ${ş}_{w,i}>{ş}_{c,i}$.^3^ The two streams are separated by a thin liquid-impermeable barrier of high thermal conductance so that the warm stream cools steadily during passage through the exchanger while the cool stream warms steadily as it accretes heat from the warm stream. Because heat energy cannot passively be transferred from regions of lower to regions of higher temperature, the efflux temperature of the warm stream must satisfy the inequality

1.1 $${ş}_{w,i}>{ş}_{w,e}>{ş}_{c,i}.$$

Similarly,

1.2 $${ş}_{w,i}>{ş}_{c,e}>{ş}_{c,i}.$$

In addition to this *counterflow* heat exchanger configuration, configurations known as *crossflow* (streams predominantly perpendicular) and *coflow* (streams predominantly parallel) exist; but they will not be discussed here.

In any era when useful energy is expensive and/or scarce, there are apt to be strong economic and/or regulatory pressures to capture the valuable heat energy, which may be of negligible worth to the system under study if it remains in the warm stream as it exits, and transfer it to some other stream where it will be useful. One measure of the quality of this heat exchange is the degree to which the warm stream can be stripped of its unneeded heat energy. Suppose that the number of moles per unit time of warm liquid transiting an adiabatic exchanger is *M*_w_ and that its molar-specific heat is *γ*_w_; then the actual rate of heat loss in transit will be just $M_{w}\gamma_{w}[{ş}_{w,i}-{ş}_{w,e}]$. Since, by equation (1.1), the warm exit temperature can never be less than the cool-stream influx temperature, the maximum loss rate is $M_{w}\gamma_{w}[\;{ş}_{w,i}-{ş}_{c,i}]$. Hence one can define a figure of merit *ε* for the exchanger as (cf. figure 1)

1.3 $$\varepsilon =\frac{\text{actual heat transfer rate}}{\text{limiting heat transfer rate}}=\frac{\left[{ş}_{w,i}-{ş}_{w,e}\right]}{\left[{ş}_{w,i}-{ş}_{c,i}\right]}<1,$$

1.3' $$=\frac{\left[{\hat{S}}_{w,i}-{\hat{S}}_{w,e}\right]}{\left[{\hat{S}}_{w,i}-{\hat{S}}_{c,i}\right]}<1$$

1.3” $$=\frac{\left[S_{w,i}-S_{w,e}\right]}{\left[S_{w,i}-S_{c,i}\right]}<1,$$

where, for any combination of subscripts, $S=\hat{S}/{\hat{S}}_{0}$; *ε* is commonly called the (thermal) ‘effectiveness’. The goal, therefore, in heat exchanger design is to make *ε* as close to 1 as is economically and/or physically practical.

In a real parallel-plate heat exchanger a common practice is to have a stack of many liquid layers separated by thin diaphragms and alternating as warm–cool–warm–cool–warm–cool, etc. This can be idealized as *N* of the unitary exchangers discussed above, but piped in parallel.

To someone educated in the physical sciences and drilled upon problems of passive heat flow in stationary (normally solid) media, the idea of effectively interchanging the heat contents of two flowing streams may well seem surprising. And, therefore, §2 is devoted to a tutorial thought-experiment which clearly shows that, in principle and for an adiabatic exchanger, such exchange is indeed possible and with thermal effectiveness approaching 1—if one has all the time in the world to wait plus material resources sufficient to lengthen the exchanger indefinitely.

However, in a real rectilinear exchanger stream of uniform cross-section *A*, half-length *L* and mean (or nominal) liquid velocity *U*, the liquid has only the nominal dwell time 2*L*/*U*, where *U* = *M*V/*A*, *M* being the molar flux rate of the liquid and V being its molar volume. Moreover, the thermal conductivity *κ* of the membrane separating the two streams will be finite, and this will restrict the amount of heat which can be transferred during that dwell time. To estimate the effects of finite *U* and *κ*, §3 is given over to a brief review of the idealized one-dimensional convection–diffusion problem to show that the effectiveness is given by equation (3.4).

Moreover, if a liquid stream within the exchanger is free of turbulence, then heat transfer from it to the other stream must in some fashion depend upon a constant D of effective transverse thermal diffusivity. To estimate this effect, it is convenient to scrap the one-dimensional idealization of §3 and to replace it with a two-dimensional idealization in which *κ* does not appear. This is done in §4 where it is shown: (i) that the resulting problem reduces to a difficult boundary value problem, which requires a novel and non-obvious analytic and numerical treatment; (ii) that, under these circumstances, the effectiveness is given by equation (11.1); and (iii) that, remarkably, this simple form is qualitatively accurate whether the liquid velocity profile across a layer of moving liquid within the exchanger is uniform (plug flow) or Hagen–Poiseuille (laminar flow).

Our analyses will show clearly that *high effectiveness, large throughput and low cost are unlikely to characterize the same exchanger: choose at most two*.

## 2. A quantized counterflow heat exchanger

Heat exchangers as manufactured and used are continuous flow devices, and to our knowledge, a rigorously quantized device has not previously been proposed. Nevertheless, quantization is a useful tool for understanding the physics of exchanger operation.

**Figure 2.**
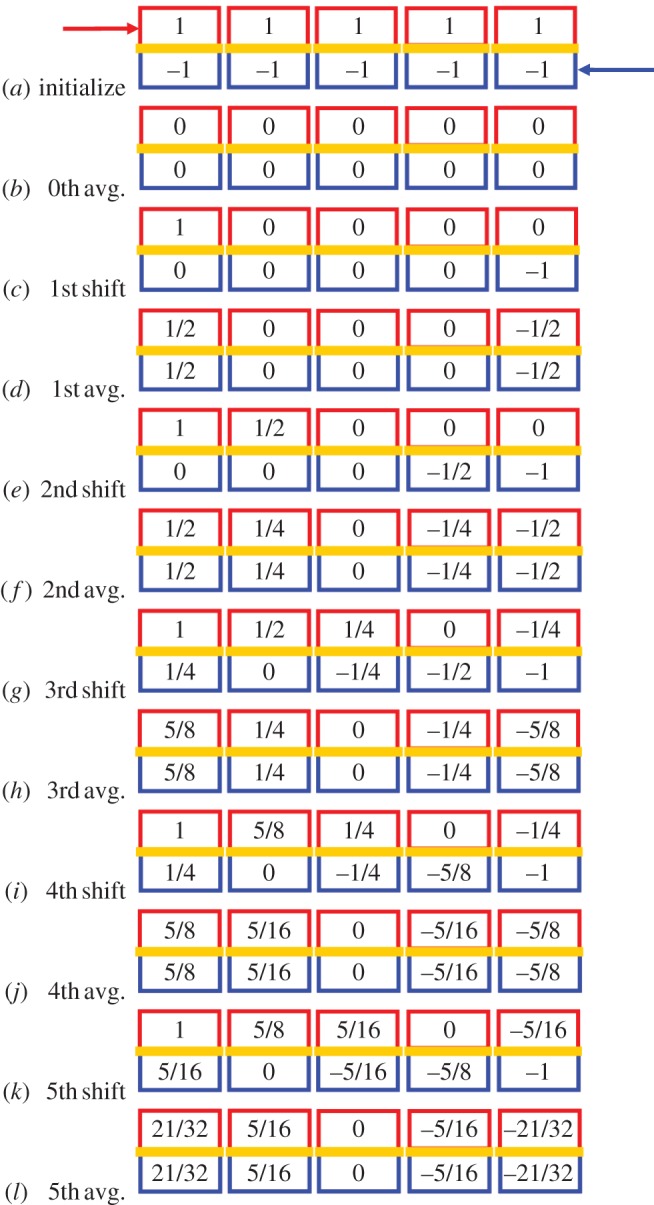
Schematic diagram of the change with time of the temperatures within a five-step, quantized, ideal, symmetric, heat exchanger. Boluses of warm liquid (red), thermally insulated on three sides, enter at the upper left at temperature +1 and progress stepwise to the right. Boluses of cool liquid (blue), thermally insulated on three sides, enter at the lower right at temperature –1 and progress stepwise to the left. But, after each step, the boluses of a vertical pair can exchange heat by way of a membrane (golden), which is thermally conducting only in the vertical direction. Each bolus is, except for heat content, assumed to be identical to all other boluses. (*a*) The warm and cool streams are initialized from the inlet ports of the heat exchanger. (*b*) The vertical pairs are permitted to equilibrate thermally, but not to exchange heat with their surroundings. (*c*) The five ‘warm’ boluses are right-shifted, with the right-most bolus being discharged at temperature 0 into the warm efflux stream, and the left-most bolus being replaced from the warm influx stream with fluid at temperature +1. The ‘cool’ bolus stream is left-shifted analogously. The steps *b* and *c* are then iterated through *d* and *e*, *f* and *g*, etc. The asymptotic temperature distribution of the vertically equilibrating quanta within the exchanger is 2/3, 1/3, 0, –1/3, –2/3, which implies an effectiveness of 5/6 ≅ 0.8333.

Suppose, therefore, that two sequences of liquid-filled packets enter the exchanger of figure 1 by stepwise displacement; they are identical except for their liquid temperatures and their synchronized antisymmetric stepping. They do *not* exhibit steady flow but instead operate in stepping mode so that discrete boluses of liquid are periodically displaced. Within the exchanger, let there be $\tilde{N}$ boluses $\left(1,2,\ldots ,\tilde{n},\ldots ,\tilde{N}\right)$ in each stream. Suppose that these isolated boluses are unstirred and thermally isolated from their surroundings, except that (as illustrated in figure 2 for $\tilde{N}=5$) the $\tilde{n}\text{th}$ bolus of each stream is in thermal contact with the *n*th bolus of the other stream. Finally, transform temperature to the ‘normalized symmetrized sensible’ s-scale described in figure 1: in this scale, the warm influx is at *S*_w,i_ = 1 and the warm efflux at 1 > *S*_w,e_ > –1; similarly the cool influx is at *S*_c,i_ = –1 and the cool efflux at 1 > *S*_c,e_ > –1. The operation cycle is:

(i) Rapidly load $\tilde{N}$ boluses from each influx stream.
(ii) Allow the $\tilde{N}$ vertically adjacent bolus warm/cool pairs to equilibrate thermally with each other.
(iii) Right shift the warm stream by one bolus, with the bolus at the left end being replaced by a fresh bolus from the influx pipe and the one initially at the right end being discharged into the efflux pipe. Analogously, left shift the cool stream by one bolus.
(iv) Allow the *Ñ* bolus warm/cool pairs to equilibrate thermally with each other.
(v) Iterate on steps (iii) and (iv).

In this fashion heat is exchanged between the warm and cool streams.

*Example:* $\tilde{N}=1$. In this case, the equilibrium temperature of the only bolus-pair is always 0. Thus, the efflux pipes fill with liquid at temperature zero. Moreover, *S*_w,e_ = 0 thereby setting *ε*_1_ = [1 – 0]/[1 – (–1)] = ½.

*Example:* $\tilde{N}=5$. This case is shown in figure 2, which illustrates the operation of the first few cycles. Further calculation would show that, as the number of cycles becomes large, the equilibrated temperature distribution (from left to right) tends towards 2/3, 1/3, 0, −1/3, −2/3; and effectiveness during steady-state exchange becomes *ε*_5_ = 5/6.

*Exploration:* $\tilde{N}$ *arbitrary*. Based upon the above two examples, one might hazard a guess that the temperature distribution at steady state has a linear temperature fall-off between influx and efflux with the difference between adjacent boluses being $2/(\tilde{N}+1)$. If this is the case, then successive boluses of the cool stream will have equilibrium temperatures of: *s*_w,influx_ = ($\tilde{N}$ + 1)/($\tilde{N}$ + 1); *s*_1_ = ($\tilde{N}$ – 1)/($\tilde{N}$ + 1); *s*_2_ = ($\tilde{N}$ – 3)/($\tilde{N}$ + 1); …; *s_n_* = ($\tilde{N}$ + 1 – 2*n*)/($\tilde{N}$ + 1); …; $s_{\tilde{N}}$ = –($\tilde{N}$ – 1)/($\tilde{N}$ + 1) = *s*_w,efflux_. This distribution is clearly invariant under the shift and average operation of the two streams and therefore is, by definition, the equilibrium distribution. Hence, an adiabatic quantized counterflow heat exchanger of $\tilde{N}$ interior boluses has effectiveness $\varepsilon_{\tilde{N}}=\tilde{N}\text{/( }\tilde{N}+1\text{) .}$ Therefore, *the asymptotic efficiency of such an exchanger is* *ε*_∞_ = 1.

The upshot of this is that, at least in a thought experiment, it is possible to exchange the heat contents of two equivalent volumes of liquid which are distinguished only by their temperatures. This violates no thermodynamic law because (ideally) no work is done in the process.

## 3. A one-dimensional continuous heat exchanger

**Figure 3.**
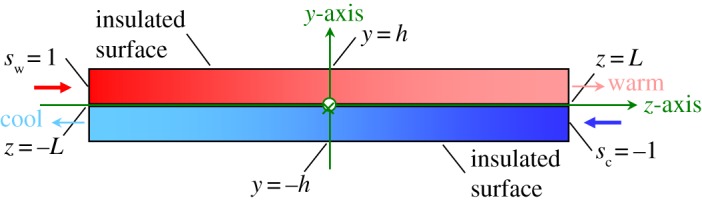
Schematic of one functional warm–cool pair of a quantized counterflow heat exchanger. The warm-pipe inlet temperature is assumed to exceed the cool-pipe inlet temperature. There is no variation in the *x*-direction, which is assumed to extend from +*d* to –*d*. All flow within the exchanger is assumed to be parallel (or antiparallel) to the *z*-axis. Heat exchange with the surroundings, except that associated with the influx and efflux streams, is presumed minor because either (i) the unit is interior to an *N*-unit sandwich (N≫1) or (ii) the outer surfaces of the unit are heavily insulated. Ideally, the fluid flow in each pipe will be steady and *z*-invariant; also the velocity profile in the *y*-direction will be symmetric about the line *y* = *h*.

Under steady operation, a one-dimensional counterflow heat exchanger (with homogeneous composition in any transverse cross-section and constant inputs) reaches an equilibrium state within which the amount of heat in any cross-sectional slab is constant; the overall geometry of such an exchanger is shown in figure 3 for the case of antisymmetric warm and cool pathways. For a thin *perfectly mixed* transverse cross-section in the warm stream^4^ [11], this heat balance is given by

3.1 $$0=MM\gamma {\hat{S}}_{0}[s_{w}(z)-s_{w}(z+\Delta z)]-\kappa \left[\frac{2d\Delta z}{2a}\right]{\hat{S}}_{0}[s_{w}(z)-s_{c}(z)],$$

where M is the molar-specific weight, *M* is the molar flow rate of the influx, *γ* the molar-specific heat of the liquid influx, ${\hat{S}}_{0}$ the symmetrized input temperature of the warm stream, *κ* the thermal conductivity of the membrane separating the two streams, 2*a* the thickness of the membrane and 2*d* the depth of the membrane in the *x*-direction. As Δ*z *→ 0, equation (3.1) becomes

3.2*a* $$0=\frac{\text{d}s_{w}}{\text{d}z}+\Omega [s_{w}-s_{c}]$$

and, analogously, the heat balance equation for the cool stream becomes

3.2*b* $$0=\frac{\text{d}s_{c}}{\text{d}z}+\Omega [s_{w}-s_{c}],$$

where the parameter Ω is given by

3.2*c* $$\Omega =\frac{\kappa d}{MM\gamma a}.$$

The appropriate boundary conditions are

3.2*d* $$s_{w}(-L)=+1$$

and

3.2*e* $$s_{c}(+L)=-1.$$

The solution of the system (3.2) is readily found by a variety of means. One which seems intuitively satisfying is to note from symmetry that *s*_c_(*z*) = –*s*_w_(–*z*), express *s*_w_(*z*) as the sum of odd and even functions, and deduce eventually from this that

3.3*a* $$s_{w}(z)=\frac{1-2\Omega z}{1+2\Omega L}$$

and

3.3*b* $$s_{c}(z)=\frac{-1-2\Omega z}{1+2\Omega L}.$$

From equations (3.3) it follows that, based upon this one-dimensional idealization, the prospective effectiveness of a counterflow heat exchanger is

3.4 $$\varepsilon_{1D}=1/2[s_{w}(L)-s_{c}(L)]=\frac{2\Omega L}{1+2\Omega L}=1-\frac{1}{1+2\Omega L}.$$

Suppose that, initially, an exchanger is designed to have 2Ω*L* = 1 and thus an effectiveness of only 50%: doubling the length gets the unit to 67%; tripling it gets 75% and making it longer still could well increase the cost prohibitively.^5^ Raising effectiveness of the exchanger by increasing Ω may also be problematic: if anything, the exchanger's owner probably would like to increase the throughput *M*, which would decrease effectiveness; *κ* and *γ* are presumably constrained by the nature of the problem; greatly decreasing the membrane thickness 2*a* would doubtlessly weaken the device structurally; and increasing the depth 2*d*, like increasing the length 2*L*, will soon produce large cost increases for small effectiveness increases. In the very high flow rate limit as 2Ω*L *→ 0, it is seen that *ε*_1D_ ∝ *L*/*M*.

In practice, counterflow exchangers are a well-developed and mature technology. In consequence, the recent literature on their one-dimensional idealization has been sparse in recent decades. However, the interested reader may wish to consult further references [12–16].

## 4. A two-dimensional continuous heat exchanger

### 4.1. General case

**Figure 4.**
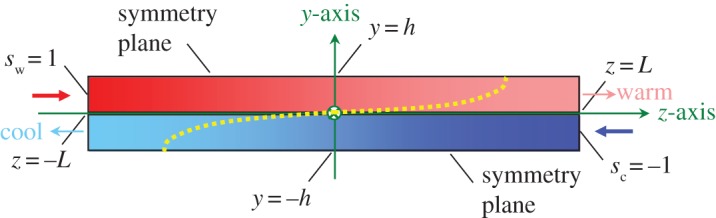
Schematic of one symmetry unit of a counterflow heat exchanger. The fluid velocity will have a peak at each symmetry plane; but there will be no heat flux across such planes. Generic profiles of velocity across the warm and cool streams of the unit are shown as yellow dots. The profile in the warm stream will be *Uf*(*υ*) while that in the cool stream is taken to be its mirror image –*Uf*(–*υ*).

Suppose now that we take our steady-state idealization of the intra-exchanger liquid streams to a different limit. Let the thickness of the membrane separating the streams become vanishingly thin (*a *→ 0) so that there will be continuity of both temperature and heat flux across the interface at *y* = 0. Assume that heat flux is (i) negligible in the *x*-direction, (ii) is overwhelmingly diffusive in the y-direction and (iii) is overwhelmingly convective in the z-direction, where the convection can be idealized as constant strictly axial flow velocity independent of transverse position. This is illustrated in figure 4, which will be recognized as a modification of figure 3. The principal differences between figures 3 and 4 are (i) that generic laminar flow profiles have been superposed upon the two channels and (ii) that symmetry planes rather than unit boundaries have been indicated. In a many-unit stack of warm–cool flow sheets, there will be *N* warm–cool flow sheet pairs (each 4 h thick) and *approximately* 2*N functional* pairs (each 2 h thick). Also, make the substitutions *y* = h*υ* and *z* = *Lζ*. Then, by repeating the formalism of any standard text on heat conduction ([17], §1.7, esp. eqn 1.7(1)) and simplifying the *x*-directed, *y*-directed and *z*-directed fluxes,^6^ that

4.1*a* $$0=\frac{\partial^{2}s_{w}}{\partial \upsilon^{2}}-\Lambda^{2}f(|\upsilon |)\frac{\partial s_{w}}{\partial \zeta },\;\;0<\upsilon <1,\;-1<\zeta <1$$

and

4.1*b* $$0=\frac{\partial^{2}s_{c}}{\partial \upsilon^{2}}-\Lambda^{2}f(|\upsilon |)\frac{\partial s_{c}}{\partial \zeta },\;\;-1<\upsilon <0,\;-1<\zeta <1.$$

where the boundary conditions are

4.1*c* $$\begin{array}{rl} & s_{w}(\upsilon ,-1)=1,\;0<\upsilon <1,\;\;\zeta =-1, \end{array}$$

4.1*d* $$\begin{array}{rl} & s_{c}(\upsilon ,1)=-1,\;-1<\upsilon <0,\;\;\zeta =1, \end{array}$$

4.1*e* $$\begin{array}{rl} & \frac{\partial s_{w}}{\partial \upsilon }=0,\;\upsilon =1,\;-1<\zeta <1, \end{array}$$

4.1*f* $$\begin{array}{rl} & \frac{\partial s_{c}}{\partial \upsilon }=0,\;\upsilon =-1,\;-1<\zeta <1, \end{array}$$

4.1*g* $$\begin{array}{rl} & s_{w}(0,\zeta )=s_{c}(0,\zeta ),\;\;\upsilon =0,\;\;-1<\zeta <1, \end{array}$$

4.1*h* $$\begin{array}{rl} \text{and}\; & \frac{\partial s_{w}}{\partial \upsilon }=\frac{\partial s_{c}}{\partial \upsilon },\;\upsilon =0,\;-1<\zeta <1, \end{array}$$

where the constant *Λ*^2^ is defined in the Symbol Table and where *f*(*υ*) is the liquid velocity profile over [0,1], assumed to be non-decreasing and of mean value 1. We note (i) that it seems reasonable to assume, from the antisymmetries of the physical problem, that

4.2 $$s_{c}(-\upsilon ,-\zeta )=-s_{w}(\upsilon ,\zeta ),\;\upsilon \geq 0$$

and (ii) that this assumption seems to accord with equations (4.1).

The information desired from the particular two-dimensional idealization of equations (4.1) and (4.2) is the effectiveness of the energy interchange as defined by

4.3*a* $$\varepsilon_{2D}=1/2[1-S_{w,e}],$$

where *S*_w,e_ is the dimensionless warm-stream energy efflux at *ζ* = 1, which is given [18] by

4.3*b* $$S_{w,e}=\int_{0}^{1}f(\upsilon )s_{w}(\upsilon ,1)d\upsilon ,$$

where *S*_w,e_ should depend solely upon the choice of *f*(*υ*) and the value of the parameter^7^ *Λ*.

Technically, the question posed above is known as a ‘steady-state conjugate-Graetz problem’, a class which has been discussed infrequently in the archival literature [19].^8^ Generalized solutions for Graetz problems related to that of our highly idealized system (4.1) have been found [20–23]. More recently, Vera & Liñán [24] have investigated the system of equations (4.1) and (4.2) numerically. Also, Quintero & Vera [25] have investigated influence of wall and diaphragm conduction effects and found that they lower effectiveness. A fairly up-to-date review of conjugate problems in heat transfer is that of Dorfman & Renner [26]. We find these solutions distinctly recondite and point out that they seem not to have considered the simple variation of effectiveness with the dimensionless variable *Λ*.

To effect a theoretical solution, the nature of *f*(*υ*) must be specified. We define it so that the *z*-directed liquid velocity over half of a warm pipe is given by *U**f*(*υ*), where *U* is the mean velocity across the pipe. We then distinguish two special cases:

(i) Constant velocity profile (i.e. plug flow). This physically unrealistic flow profile
  4.4*a* $$f_{i}(\upsilon )=0,\;0=\upsilon$$
  and
  4.4*b* $$f_{i}(\upsilon )=1,0,\upsilon \leq 1$$
  might be relatively tractable analytically.
(ii) Parabolic velocity profile (i.e. Hagen–Poiseuille flow). This is what one normally thinks in terms of for flow between stationary planar surfaces at Reynolds numbers below 50.
  4.5*a* $$\begin{array}{rl} & f_{\mathrm{ii}}(\upsilon )=3[\upsilon -1/2\upsilon^{2}],0\leq \upsilon \leq 1, \end{array}$$
  4.5*b* $$\begin{array}{rl} & f_{\mathrm{ii}}(1)=\frac{3}{2} \end{array}$$
  4.5*c* $$\begin{array}{rl} \text{and}\; & f_{\mathrm{ii}}(\upsilon )=1@\upsilon =1-\sqrt{1/3}=0.4226, \end{array}$$

The plug flow problem might be analytically tractable, but would seem to require extreme turbulent flow to approximate its existence, thereby vitiating the convection diffusion model. On the other hand, recent computational fluid-dynamics modelling of counterflow exchangers suggests that the flow profile could in practice be laminar [27], thereby favouring a parabolic profile. The reasonableness of this finding can be seen by noting that Hagen–Poiseuille flow in a planar channel is expected to be stable when the Reynolds number ***R*** = 2hU*ρ*/*η* ≤ 2500 [28] but to deteriorate into at least vortex shedding if there are obstacles or discontinuities [29]. For a thin water-filled channel with *U *∼ 0.1 m s^–1^, h ∼ 0.005 m, *ρ* = 1000 kg m^–3^ and *η* = 0.001 Pa s, ***R*** ∼ 1000, a situation in which laminarity should obtain. On the other hand, plumbing connections at input and output might induce modest tumbling that would scramble the temperature distributions.

Therefore, even though it may not be entirely relevant, our development will begin with plug flow for which one approximate solution will be presented. Then we shall tackle Hagen–Poiseuille flow for which only numerical data will be presented. Reassuringly, there is excellent qualitative agreement between the two. Both developments demonstrate that there is significant difference between the predictions of one-dimensional theory and those of the two-dimensional theory.

### 4.2. Plug flow

Because we could find no simple straightforward solution to (4.1), (4.2), (4.3) for plug flow, we opted for the approximate treatment of appendix A. It gives the effectiveness as a function of *Λ*^9^:

4.6 $$\varepsilon =\frac{\tanh\Lambda }{\Lambda }.$$

To our knowledge, this remarkably simple approximation has not previously been published. Moreover, it reveals that effectiveness can be simply gauged using a single dimensionless constant.

### 4.3. Hagen–Poiseuille flow

**Figure 5.**
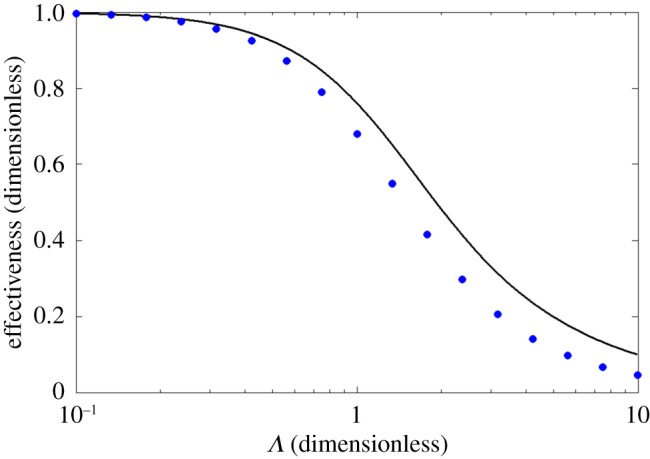
Comparison of first order Taylor series solutions of the system (equation (4.1)) for the thermal effectiveness *ε* : equations ((4.6) ≡ (A.8)) for plug flow is plotted as a solid black line; overlying this is a sequence of blue dots generated for discrete values of *Λ*: these data, for Hagen–Poiseuille flow, were obtained using the wholly numerical FDTD method described in appendix B. For *Λ* ≤ 1, the two treatments yield similar results.

In our hands, *simple* analytic solutions to neither the parabolic Hagen–Poiseuille profile nor various approximations of it were found, although closed-form solutions to equation (A.5) *do* exist for a parabolic profile [24,30]. We concluded that a numerical model of the geometry of the previous section, only with a Hagen–Poiseuille flow profile, would be an interesting test of the approximate solution of appendix A. To this end, we obtained a plot of effectiveness versus *Λ* by means of an FDTD (finite-difference time-domain) approach; our finite difference algorithm is explained in appendix B, and our results are shown in figure 5. This figure shows that: (i) the plug flow and Hagen–Poiseuille (HP) flow models for effectiveness have qualitatively similar behaviours over the entire *Λ*-range [0.1,10], only with HP-flow showing qualitative superiority everywhere; (ii) that they agree acceptably well from *Λ* ∼ 0 out to *Λ* ∼ 1 and (iii) that, for higher values of *Λ*, the expected effectiveness is so low as to render irrelevant the precision of estimate effectiveness predicted by either model becomes so low as to discourage use of exchangers operating in that high *Λ*-range.

## 5. Discussion and conclusion

There are at least four things that one might ask of a counterflow heat exchanger. The first is that the passage of the exchanger liquids through it be swift so that much stored heat becomes available to be exchanged; this will be roughly indexed as [2*h*d*U*]. The second is that the cost the materials for the exchanger be small; this will be indexed as the plate area of a channel separator [4d*L*]. The third is that the diffusion velocity in the *υ*-direction be large so that heat exchange between the two streams is enhanced; this will be indexed as [D/2h]. This then yields

5.1 $$\Lambda^{2}=\frac{\left[2h\;dU\right]}{\left[4\;dL][D/2h\right]}=\frac{h^{2}U}{LD}.$$

And the last (fourth) thing that might be asked is that *Λ* be small so that the effectiveness approaches 1−. We have here a *quadrilemma*! And it seems impossible to get everything desired.

First, increasing the throughput increases *Λ*, thereby decreasing effectiveness.

Second, decreasing the materials cost increases *Λ*, thereby decreasing the effectiveness.

Third, increasing the diffusion velocity in the *υ*-direction decreases *Λ*, thereby increasing effectiveness.

Fourth, decreasing *Λ* to < ½ will raise the effectiveness to >0.94. And, by equation (5.1), this might be accomplished by raising D and/or lowering *h*.

The thermal diffusivity of unstirred water is approximately 140 × 10^−9^ m^2 ^s^−1^, while that of a high temperature heat transfer liquid such as Duratherm 630 is approximately 77 × 10^−9^ m^2 ^s^−1^. Switching, for example, to molten sodium would up the thermal diffusivity by a factor of roughly a thousand-fold to approximately 65 × 10^−6^ m^2 ^s^−1^, but at the cost of a daunting increase in operating complexity. Alternatively, for a eutectic mixture of sodium and potassium nitrates, much used in concentrated solar power installations, the thermal diffusivity is a modest approximately 170 × 10^−9^ m^2 ^s^−1^ [31].

The real-world work-around, which seems to have bypassed the theoretical limitations of static thermal diffusivity, is a deliberately induced local turbulence superposed upon to the bulk motion of the heat transfer liquids^10^: seemingly, this produces an *effective* diffusivity large enough to force the thermal impedance between the warm and cool streams towards the thermal resistance of the thin corrugated metal plates separating these streams. This strategy appears to have been followed the past 30 years [32,33]. And it has led to heat exchangers roughly the size of a pulpit Bible and costing less than 2 k$ apiece, which are able to sustain heat transfer rates on the order of 440 kW [11]. These are not expensive hand-crafted luxuries: they are a mass-produced commodity!

Nevertheless, conversion of solar-thermal energy to and fro between stored heat and electricity still seems challenging. It has yet to been demonstrated cost-effectively at long-term utility-scale levels.

A comparison of the results of §§3 and 4 with a typical text on heat exchangers [4,34] will show that our notation is different, that our presentation is different and that our focus is different. This is because we are less interested in the hugely important practicalities of specifying, plumbing or using heat exchangers than we are in delivering to scientists from other fields an understanding how practical exchangers work and in what directions their theoretical limitations may lie.

Finally, remember that utility-scale storage and release of electric power presumably must take place at multi-megawatt levels. Endeavouring to store electrical energy as heat, which can then be converted to electricity, is therefore likely to require heat exchangers that operate at the megawatt level. Such exchangers now are commonplace and can be added as needed at a cost of around 5 dollars per kilowatt capacity (plumbing parts, labour and bulk liquid storage not included). But the small details of such generation, conversion and storage are as yet fluid because the structuring of America's smart resilient grid of the future (together with its component microgrids) is only now commencing.^11^

In conclusion, this essay is intended as a tutorial paper, where originality is not necessarily a *sine qua non*. However, we believe the quantized heat exchanger to be new. Further, we are unaware of the occurrence elsewhere of the formula ‘NTU = 2Ω*L*’; but the literature is vast, and we make no claim. Further, we believe the effectiveness formula of equation (4.6) to be original.

## Footnotes

2 One gigawatt-day = 1.00 GWd = 86.4 TJ. This is approximately equal to energy of combustion of 14 000 bbl of petroleum, where one barrel of oil equivalent has been defined to be 5.8 × 10^6^ Btu ∼ 6.12 GJ.

3 The nomenclature for these flows is admittedly complicated. The reader is encouraged to consult table 1 and figure 1 for further details.

4 Perfect transverse mixing, of course, cannot readily be achieved but may perhaps be approximated by turbulent flow. Turbulence will also flatten the velocity profile transverse to the flow. In practice, the diaphragm separating the two streams may be corrugated to *promote* turbulence and maximize the temperature gradient across the diaphragm (e.g. [11]).

5 Engineers familiar with symmetric counterflow exchangers will recognize 2Ω*L* as homologous with the NTU (number of heat transfer units) of the exchanger. This relationship is explored further in Note E of the electronic supplementary material, where it is shown that the exchanger literature is expressed overwhelmingly in terms of NTU, often without explicit regard for fluid flow regimes within the exchanger or the relationship between NTU and the geometric parameters, constitutive parameters and flow velocity.

6 **Specifically**: (i) The *z*-directed flow sheets are presumed to extend from −*d* to +*d* in the *x*-dimension, where *d* is so large that the temperatures are without significant variation in the *x*-direction. (ii) Convection is assumed to occur only in the *z*-direction. (iii) As there is no convection in the *y*-direction, the *y*-directed heat flux is diffusive only. (iv) It is presumed that heat transport in the *z*-direction is predominantly convective and lacks a significant diffusive component, except in the negligibly thin boundary layers at *y *= −2*N*h, −(2*N* − 2)h, … −2h, 0, 2h, … (2*N* − 2)h, 2*N*h.

7 The average temperature might have been chosen in equation (4.3*b*) rather total energy transfer (i.e. without the integrand weighted by *f*(υ)) because in practice the inlet and outlet temperatures one measures are a rough sort of average: use of in-stream sensors, localized turbulent mixing and possible swirling in the exchanger's plumbing could serve to add the appearance of greater accuracy than the experimental reality might support.

8 In [19], ‘double’ is used rather than ‘conjugate’; this is rare. Also in use is ‘conjugated’.

9 The relationship between *Λ* and the NTU (number of heat transfer units) of the exchanger is explored further in appendix C.

10 This appears to be accomplished by the dual strategy of increasing the Reynolds number and corrugating the plates to promote tumbling [32].

11 The elevated heat transfer rates that characterize counterflow parallel-plate exchangers seemingly depend upon increased *effective* thermal diffusivity associated with transverse turbulence within the *two* flowing media. If heat transfer in one or both of the media depends upon thermal diffusivity *sensu stricto*, as for example in a ground-source heat pump where there is stationary soil surrounding a pipe bearing moving thermal fluid, the rate of transfer can drop significantly.

## Data accessibility

Simple numerical modelling was used throughout. The numerical model of appendix B was run on Matlab; this program has been uploaded to Dryad, as has a short set of instructions for its use: https://doi.org/10.5061/dryad.2n0v8 [35].

## Appendix A

**A.1. Approximate solution by Taylor series of the system (4.1), (4.2), (4.3)**

Physically, the function *s*_w_(*υ*,*ζ*) should be well behaved about (0,0); and, therefore, a well-behaved expansion of the form

A 1 $$s_{w}(\upsilon ,\zeta )=s_{0}(\upsilon )+\zeta s_{1}(\upsilon )+1/2\zeta^{2}s_{2}(\upsilon )+2(\zeta^{3})$$

should exist. When (A 1) is substituted into (4.1*a*) and terms in *ζ*^p^ are satisfied individually, it turns out that

A 2 $$s_{0}(\upsilon )=\text{unspecified},\;s_{1}(\upsilon )=\frac{1}{\Lambda^{2}f(\upsilon )}s_{0}^{\prime \prime }\;\text{and}\;s_{2}(\upsilon )=\frac{1}{\Lambda^{2}f(\upsilon )}s_{1}^{\prime \prime },$$

where ′ = ∂/∂*υ*. It then follows from (A 1) that

A 3*a* $$s_{w}(\upsilon ,\zeta )=s_{0}(\upsilon )+\zeta \frac{1}{\Lambda^{2}f(\upsilon )}s_{0}^{\prime \prime }(\upsilon )+2(\zeta^{2})$$

and

A 3*b* $$s_{c}(\upsilon ,\zeta )=-\left[s_{0}(-\upsilon )-\zeta \frac{1}{\Lambda^{2}f(|\upsilon |)}s_{0}^{\prime \prime }(-\upsilon )+2(\zeta^{2})\right].$$

Focus upon (A 3*a*) and assume plug flow so that *f*(*υ*) = 1. Next observe that a possible trial solution for *s*_0_(*υ*) could be

A 4 $${\;}_{\left[\text{trial}\right]}s_{0}(\upsilon )=A+C\text{cosh}\;\Lambda \upsilon +S\text{sinh}\;\Lambda \upsilon ,$$

where A, C and S are locally defined constants yet to be determined. This then gives

A 5 $${\;}_{\left[1\right]}s_{w}(\upsilon ,\zeta )=A+(1+\zeta )(C\text{cosh}\;\Lambda \upsilon +S\text{sinh}\;\Lambda \upsilon ).$$

Application of the boundary conditions (4.1*c*) and (4.1*e*) then yields

A 6*a* $${\;}_{\left[1\right]}s_{w}(\upsilon ,\zeta )=1+(1+\zeta )C[\text{cosh}\;\Lambda \upsilon -\text{tanh}\;\Lambda \;\text{sinh}\;\Lambda \upsilon ].$$

By equation (4.2) this gives us the cool stream approximation

A 6*b* $${\;}_{\left[1\right]}s_{c}(\upsilon ,\zeta )=-\{1+(1-\zeta )C[\text{cosh}\;\Lambda \upsilon +\text{tanh}\;\Lambda \;\text{sinh}\;\Lambda \upsilon ]\}.$$

We shall proceed by choosing choose C = –1 to make the two approximations (A 6*a*) and (A 6*b*) agree at (0,0), the point of antisymmetry, thereby satisfying equation (4.2). Hence,

A 7*a* $${\;}_{\left[1\right]}s_{w}(\upsilon ,\zeta )=1-(1+\zeta )[\text{cosh}\;\Lambda \upsilon -\text{tanh}\;\Lambda \;\text{sinh}\;\Lambda \upsilon ]$$

and

A 7*b* $${\;}_{\left[1\right]}s_{c}(\upsilon ,\zeta )=-\{1-(1-\zeta )[\;\text{cosh}\;\Lambda \upsilon +\;\text{tanh}\;\Lambda \;\text{sinh}\;\Lambda \upsilon ]\}.$$

It follows from equation (3.4) that the flow of heat is down the *υ*-axis at all points within the exchanger streams; correspondingly, the flow of heat is up the *ζ*-axis at all points within the exchanger streams. All this is in accordance with physical intuition. Finally, inspection of equation (A 7) reveals continuity of temperature across *υ* = 0, but failure of flux continuity except at the antisymmetry point.

The temperature of the warm stream at its efflux port becomes

A 7*c* $${\;}_{\left[1\right]}s_{w}(\upsilon ,1)=1-2[\text{cosh}\;\Lambda \upsilon -\text{tanh}\;\Lambda \;\text{sinh}\;\Lambda \upsilon ],$$

which, by equations (4.3), yields

A 8 $$\varepsilon =\frac{\text{tanh}\;\Lambda }{\Lambda }.$$

## Appendix B

**B.1. Finite-difference time-domain solution of the parallel-plate exchanger for Hagen–Poiseuille flow**

It follows from the basic theory of heat conduction in a rectilinear stream of moving liquid that the dimensionless unsteady analogue of equations (4.1) is^12^

B 1 $$\frac{\partial s_{w}}{\partial \tau }=\frac{\partial^{2}s_{w}}{\partial \upsilon^{2}}-\Lambda^{2}f(\upsilon )\frac{\partial s_{w}}{\partial \zeta },\;-1<\upsilon <1,\;-1<\zeta <1.$$

The FDTD solution proceeds in a simply programmed series of steps. First, discretize each axis of the square space into 2*B* intervals to define (2*B* + 1) × (2*B* + 1) discrete points at which *s*_w_ must be found. Second, populate the (2*B* – 1) × (2*B* – 1) interior points using the plug flow approximation of equation (A 7). Third, specify the boundary points using the boundary conditions. Fourth, compute ∂*s*_w_/∂*τ* at each interior point forming a matrix, multiply this matrix by an appropriate *δτ* and add this product to the existing *s*_w_-matrix, forming an updated *s*-matrix.^13^ Now, iterate to convergence on steps 3 and 4. Of course, this procedure worked poorly for a number of reasons, which we shall enumerate below.

The boundary conditions at *υ* = ±1 were specified in terms of ∂*s*_w_/∂*u* and ∂*s*_c_/∂*u* (Neumann condition) rather than *s*_w_ and *s*_c_ (Dirichlet condition). This was resolved easily by setting *s*_w_(1,*n*) = *s*_w_(2,*n*) and *s*_c_(2*B*,*n*) = *s*_c_(2*B* + 1,*n*).

The boundary conditions at *ζ* = ±1 were mixed, half of each being unspecified.

Physically this lack of specification is immaterial because the unspecified portions were exit ports for the moving thermal liquid. Mathematically, applying a zero normal derivative Neumann condition seemed associated with poor convergence. We, therefore, extrapolated temperature from the interior to obtain Dirichlet conditions for the unspecified surfaces:

B 2*a* $$s_{w}(m,1)=s_{w}(m,2)+[s_{w}(m,2)-s_{w}(m,3)],\;(B+1\leq m\leq 2B)$$

and

B 2*b* $$s_{w}(m,2B+1)=s_{w}(m,2B)+\text{[}\;s_{w}(m,2B)-s_{w}(m,2B-1)\text{]}\;,\;(B+2\leq m\leq 2B).$$

This yielded results that we trusted.

Despite the generally good advice [36] that the ‘orders of the discrete derivatives should be equal to the orders of the corresponding derivatives', we had better results if we used second-order derivatives for both spatial derivatives at interior points of the lattice:

B 3*a* $$\frac{\partial s_{w}}{\partial \zeta }≅[s_{w}(m,n+1)-s_{w}(m,n-1)]\frac{B}{2}$$

and

B 3*b* $$\frac{\partial s_{w}}{\partial \upsilon^{2}}≅[s_{w}(m-1,n)-2s_{w}(m,n)+s_{w}(m+1,n)]B^{2}.$$

Finally, we had to choose *δτ* to carry out the iteration. Since |*s*_w_| < 1 at all interior points of the domain and precision to ±0.0002 is entirely adequate, we absolute-valued the ∂*s*_w/_∂*τ*-matrix at each iteration and divided 0.0001 by its largest element to find a very conservative value of *δτ* for that iteration.

Computation for *Λ* = 10 and 10^5^ time steps yielded a thermal effectiveness of 0.1586 for *B* = 5, of 0.1396 for *B* = 10, of 0.1332 for *B* = 20, of 0.1305 for *B* = 50 and of 0.1300 for *B* = 100. Calculation for *B* = 100 and 10^5^ time steps yielded convergence of the thermal effectiveness to ±0.0002 for the values of *Λ* plotted in figure 5 as blue dots. The conclusions to draw from figure 5 are (i) that all tanh *Λ*/*Λ* and FDTD plots lie within about 10% of one another over the *Λ*-range (0.1,6.0) and (ii) that beyond the upper end of this range the marginal gain of thermal effectiveness is so low one would hesitate to recommend such a device.

## Appendix C

**C.1. Expressing the number of (heat) transfer units in terms of the physical parameters 2Ω*L* or *Λ***

It is common in practical heat transfer engineering to express the effectiveness of a symmetric counterflow heat exchanger in terms the abstract quantity NTU (number of heat transfer units) as ([34], table 3.6)

C 1 $$\varepsilon =\frac{\text{NTU}}{1+\text{NTU}}.$$

From equation (3.4), it is immediately seen that for the ideal one-dimensional heat exchanger of §3, NTU = 2Ω*L* ∝ *L*/*M*. Consider now setting NTU = *p*(*p* = 0,1,2, …) and increasing it stepwise from zero to see how fast the effectiveness increases step by step. The increase from the (*p *– 1) step to the *p*th is then

C 2 $$\omega_{p-1,p}=\frac{1}{p(1+p)}.$$

The sequence of steps is: ^1^/_2_, ^1^/_6_, ^1^/_12_, ^1^/_20_, ^1^/_30_, ^1^/_42_, ^1^/_56_, ^1^/_72_, … ; and, if each unit increase of NTU is as costly as the one preceding, it is seen that the marginal cost of increasing effectiveness soon becomes quite daunting.

Comparison of (C.1) with (4.6) shows that

C 3*a* $$\begin{array}{rl} \text{NTU} & =\frac{\text{tanh}\;\Lambda }{\Lambda -\text{tanh}\;\Lambda }, \end{array}$$

C 3*b* $$\begin{array}{rl} & >3\Lambda^{-2},\;\Lambda \to 0, \end{array}$$

C 3*c* $$\begin{array}{rl} \text{and}\; & >\Lambda^{-1},\;\Lambda \to \infty . \end{array}$$

These results could be interpreted as meaning that NTU, while highly useful for both intuitive and pedagogical purposes, could be and has proved difficult to predict accurately in practice.^14^
